# Design and Implementation of an Integrated Control Scheme for GaN-Based Multiple Power Converters

**DOI:** 10.3390/mi14040833

**Published:** 2023-04-11

**Authors:** Chao-Tsung Ma, Bing-Hong Yao

**Affiliations:** Applied Power Electronics Systems Research Group, Department of EE, CEECS, National United University, Miaoli City 36063, Taiwan

**Keywords:** renewable energy (RE), distributed generation (DG), micro-grid, wide ban gap (WBG) semiconductor, power converter

## Abstract

In response to the rapid changes in the international energy environment, developing renewable energy (RE)-based distributed generation (DG) and various smart micro-grid systems is crucial for creating a robust electric power grid and new energy industries. In this aspect, there is an urgent need to develop hybrid power systems suitable for coexistent AC and DC power grids, integrated by high-performance wide ban gap (WBG) semiconductor-based power conversion interfaces and advanced operating and control strategies. Due to the intrinsic feature of variation in RE-based power generation, the design and integration of energy storage devices, real-time regulation of power flow, and intelligent energy control schemes are key technologies for further promoting DG systems and micro-grids. This paper investigates an integrated control scheme for multiple GaN-based power converters in a small- to medium-capacity, grid-connected, and RE-based power system. This is the first time that a complete design case demonstrating three GaN-based power converters with different control functions integrated with a single digital signal processor (DSP) chip to achieve a reliable, flexible, cost effective, and multifunctional power interface for renewable power generation systems is presented. The system studied includes a photovoltaic (PV) generation unit, a battery energy storage unit, a grid-connected single-phase inverter, and a power grid. Based on system operation condition and the state of charge (SOC) of the energy storage unit, two typical operating modes and advanced power control functions are developed with a fully digital and coordinated control scheme. Hardware of the GaN-based power converters and digital controllers are designed and implemented. The feasibility and effectiveness of the designed controllers and overall performance of the proposed control scheme are verified with results from simulation and experimental tests on a 1-kVA small-scale hardware system.

## 1. Introduction

Since the industrial revolution in the 18th century, human beings were using fossil fuels in a large scale, so the emissions of greenhouse gases, such as carbon dioxide, were increasing, which aggravated the greenhouse effect and caused global warming to become more serious. Fossil fuels face problems such as rapid declines in reserves and greenhouse gas emissions, highlighting the importance of developing alternative energy sources. Therefore, low-carbon energy technologies, such as nuclear energy and RE systems, became the targets of development worldwide. Although nuclear energy belongs to the low-carbon energy category, as REs are, it still has problems such as safety and nuclear waste issues. Therefore, countries around the world may need to re-examine the positioning of nuclear power generation. On the other hand, RE-based energy systems, mainly including wind energy, solar energy, hydropower, biomass energy, and geothermal energy systems, highly increased in recent years, among which wind and solar energy systems are the mainstream. According to the projection in International Energy Outlook 2023, the share of wind and solar energies in RE generation will increase to around 75% by 2050 [1]. The advantages of RE generation include: (1) inexhaustible and widely distributed; (2) low carbon emissions, no fuel, no waste, and no pollution during generation; and (3) it has little impact on the environment and is extremely environmentally friendly. However, the disadvantages of RE generation include: (1) intermittent and susceptible to weather conditions; (2) unpredictable; (3) low conversion efficiency; and (4) low power density.

**Figure 1:**
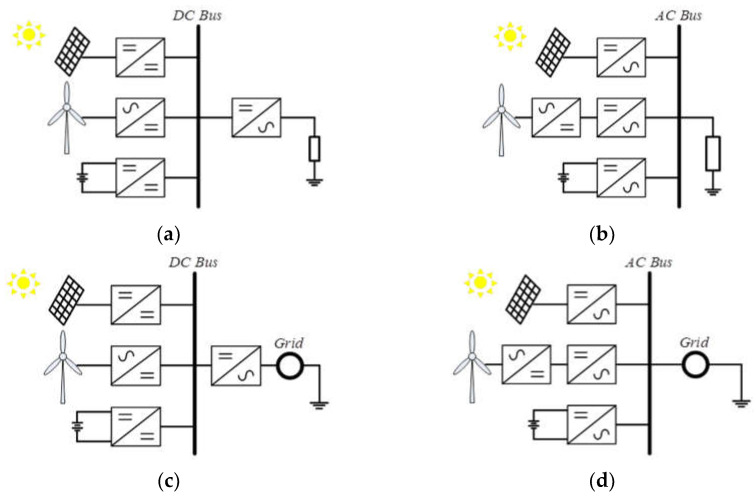
Hybrid RE power generation systems: (**a**) DC bus-based island system; (**b**) AC bus-based island system; (**c**) DC bus-based grid-connected system; and (**d**) AC bus-based grid-connected system.

The unstable power generation features in RE-based systems will greatly impact the power quality and reliability of conventional power systems. Therefore, a feasible RE power generation system is combined with a variety of RE sources and energy storage devices, which can provide minimum fluctuations in power generation to achieve an acceptable level of voltage stability. In practice, hybrid RE power generation systems can be divided into two types according to whether they are connected with the grid, i.e., island and grid-connected systems [2]. These two types of systems can be further divided into four configurations according to whether the system’s operation is based on a DC or an AC bus, as shown in Figure 1. 

Grid-connected systems are suitable for areas with adequate grid facilities. Their advantage is that when RE generation is sufficient, it can be directly supplied to the load and charge the energy storage device, and the excess power can be fed back to the grid; when the RE generation is insufficient, the energy storage device is then used to supply the load, but if the SOC of the energy storage device is also insufficient, the utility power supply is used to supply the load and charge the energy storage device, forming an uninterruptible power supply (UPS) system [3,4,5,6,7,8]. The authors of [6] proposed a control model based on a grid-connected hybrid PV power generation system to optimize power distribution, minimize the cost of purchasing electricity from the grid, and maximize the sales profit of RE. The research paper [7] discussed power converter control strategy of grid-connected systems. In [8], a new configuration with the fewest number of converters for grid-connected wind/PV power generation systems is proposed. The study performs system simulations with MATLAB/Simulink, and finally verifies that the proposed configuration can effectively reduce conversion loss and improve system performance. The use of grid-connected systems requires special attention to the output variation characteristics of RE, which tend to greatly reduce the stability of a power grid. Therefore, there are many papers working on power smoothing technologies and methods for RE-based systems [9,10,11,12,13,14,15,16]. Previous research [9,10] used energy storage batteries and supercapacitors to form a hybrid energy storage device and propose related energy management strategies to smooth the system’s power flow, thereby improving system efficiency. The authors of references [11,12] adopted a flywheel energy storage device and used moving average and linear programming to optimize the operation of the system. Experimental results show that the output power fluctuation of RE-based units can be effectively reduced. In the aspect of controller design, peak shaving, fuzzy neural networks (NN), adaptive control, and short-term prediction data to achieve power, smoothing objectives were fully discussed in [13,14,15,16].

In addition to improving the output power fluctuation of RE-based power generation systems, improving energy efficiency is also an important issue. In the case of PV generation systems, the most effective way to increase power generation efficiency is using maximum power point tracking (MPPT) control strategies. In the research paper [17], the pros and cons of three MPPT algorithms currently widely used, i.e., perturb and observe, incremental conductance, and open-circuit voltage are evaluated. Conventional MPPT technologies are mostly designed on the premise that the system has only one maximum output power point. However, in practical applications, the PV generation system is very likely to encounter partial shading. This can result in multiple local maxima in the system, in which case, conventional MPPT techniques cannot track the global maximum, resulting in considerable energy loss. The PV power generation characteristics under partial shading conditions and an improved MPPT technology based on evolutionary algorithms and swarm intelligence were investigated in [18]. It should be noted that the development of the energy storage device has a key influence on hybrid RE-based power generation systems, and the quality of charging/discharging technology has a considerable impact on the life of the energy storage device. Conventional charging methods include constant voltage and constant current charging methods [19]. The more popular charging methods proposed in recent years include constant voltage–constant current charging, pulse current charging, and sinusoidal ripple current charging methods. The authors of [20] proposed an inductive constant voltage–constant current charging method for lithium batteries in electric vehicles (EVs). This method has high efficiency and high reliability, and the experimental results show that the charging efficiency can be as high as 96.1%. The authors of [21] proposed a positive–negative pulse current charging method based on a bidirectional DC-DC converter and constructed a theoretical model. A novel sinusoidal ripple current charging method with simple configuration, low cost, simple control, and high efficiency features was presented in [22]. Experimental results show that the maximum charging efficiency can reach 95.95%. In addition, many experts and scholars proposed relatively new charging control strategies. The authors of [23] utilized the gray prediction technology to propose a smart charging control strategy for a lithium-ion battery charging system, which can increase the charging speed by 23% and the efficiency by 1.6% compared with general constant voltage–constant current charging systems. Based on the fuzzy control theory, the authors of [24] proposed an active SOC controller and proved that the charging speed can be increased by 23%. 

**Figure 2:**
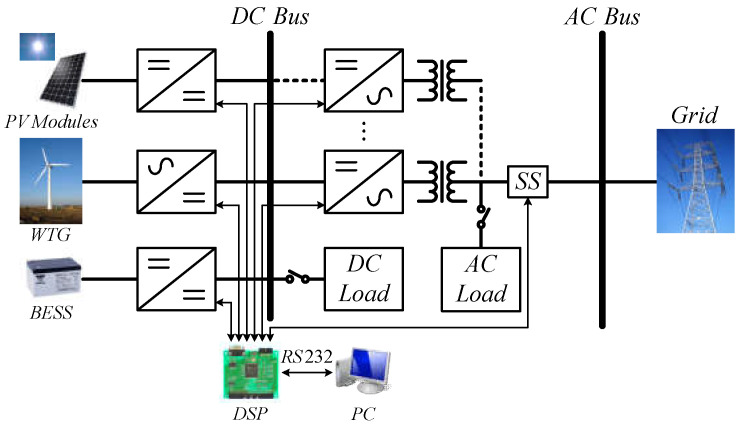
A grid-connected RE power generation system with an integrated control configuration.

To achieve high-performance RE-based power generation systems, there are still many research themes. Some typical engineering technologies and controller design issues were discussed in [25,26,27,28,29]. Potential topics include the smart integration of various RE-based power generation units, energy storage devices, power grid, and system optimization with coordinated control schemes. In addition, developing smart RE-based power generation systems with advanced power converters and advanced control strategies to achieve high system efficiency and high operation flexibility became a popular research topic in the power and energy research field. In view of this, the focus of this paper is to present the design details of an integrated control scheme for grid-connected RE-based power generation systems with WBG semiconductor-based power conversion interfaces. The overall system configuration investigated is shown in Figure 2. The proposed grid-connected RE-based power generation system uses multiple gallium nitride (GaN)-based power converters, optimally integrated with a coordinated control scheme using a programmable DSP. For demonstration purposes, two typical operating modes are designed according to the SOC of the energy storage device and the system operating condition in various application scenarios.

## 2. System Configuration and Operating Mode Planning

**Figure 3:**
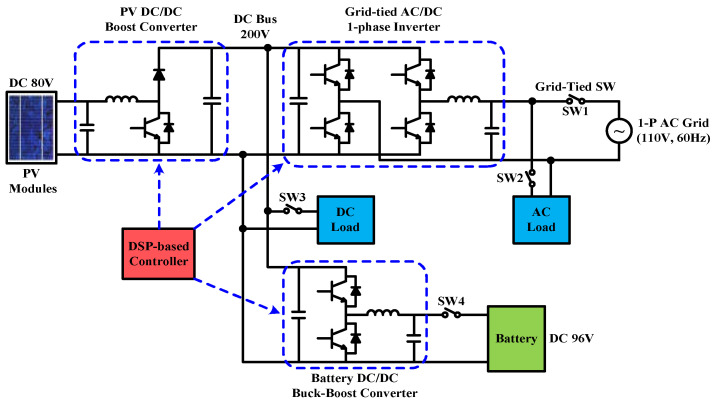
Grid-connected RE power generation system configuration.

The configuration of the grid-connected RE power generation system developed in this paper is shown in Figure 3. A 200 Vdc to 110 Vac single-phase inverter connects the system to the grid. Eight batteries are connected in series to construct a 96 V battery bank (working as an energy storage device). A bidirectional buck–boost DC/DC converter controls the charge/discharge current of the battery bank. In addition, considering the physical size of the PV modules and the hardware test requirement, a PV emulator is selected as the RE source, and a PV-boost DC/DC converter is used as the MPPT controller. Finally, a DC load can be connected to the DC bus or an AC load can be connected to the AC terminal to simulate possible DC and AC loads of the power system in various application scenarios.

In practice, a number of system operating modes can be designed for the proposed system shown in Figure 3. These operating modes mainly take into account the SOC of the energy storage device, PV power generation, grid demand, fluctuation of PV output power, and different time-based electricity prices set by the power company. In this paper, the overall system performance in two typical operating modes, i.e., PV power smoothing mode and peak power regulation mode are demonstrated with key power flow control results.

### 2.1. Operating Mode 1: PV Power Smoothing Mode

**Figure 4:**
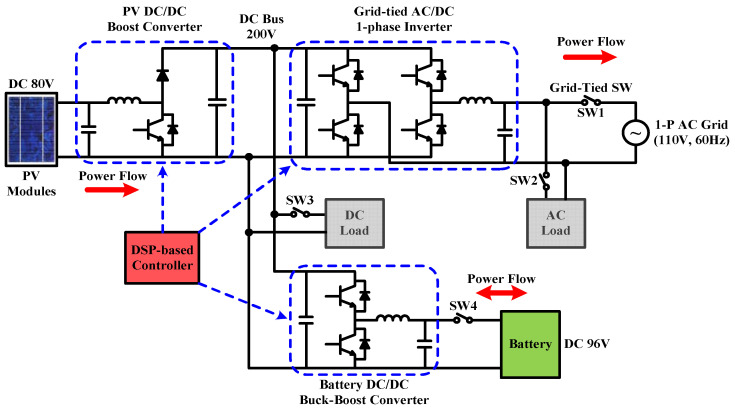
Operation and the key power flow in mode 1.

PV power generation is susceptible to cloud movement, solar eclipse, shading, etc., and may have large power fluctuations in a short period of time when MPPT is functioning. If power fluctuations are directly injected into the grid, it will greatly impact the reliability and stability of the grid. Therefore, power smoothing technology is much required. Due to the need to immediately charge/discharge the battery to smooth out the fluctuation of PV generation, this mode requires a moderate level of battery SOC. In this mode, SW1 and SW4 in Figure 3 need to be on. Based on the level of battery SOC, and PV power, the battery charge/discharge command is instantaneously calculated, and the charge/discharge control is performed by the buck–boost converter. The operation and power flow are shown in Figure 4.

### 2.2. Operating Mode 2: Peak Power Regulation Mode

**Figure 5:**
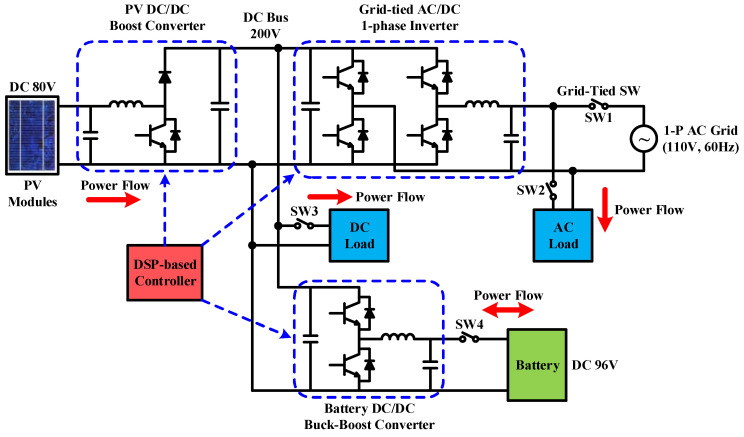
Operation and power flow in mode 2.

Load power demand may vary according to the time of the day and different seasons. Therefore, in order to reflect different electricity rates for different time periods, different electricity prices were charged. Normally, the price is high during a peak period (season) and low during an off-peak period (season). For users, it is beneficial to move peak power usage to off-peak periods. From a power company’s point of view, this can reduce peak loads causing the need for building new power plants and thus reduce power costs. However, even if power-using habit is adjusted, users cannot completely avoid power usage during peak periods. Using other power sources can help avoid using the more expensive utility power during peak periods. The main operation requirement of this mode is a sufficient battery SOC, and it is even better if PV power generation is also available. In this case, the power demand of the load can be primarily supplied by PV power generation. The energy storage device helps supply the load or absorbs excessive generation. In this mode, SW1 and SW4 in Figure 3 need to be on, and the statuses of SW2 and SW3 depend on the power demand. The operation and power flow are shown in Figure 5.

## 3. Quantitative Design of Controllers

### 3.1. Quantitative Design of Controllers for Single-Phase Inverter

**Figure 6:**
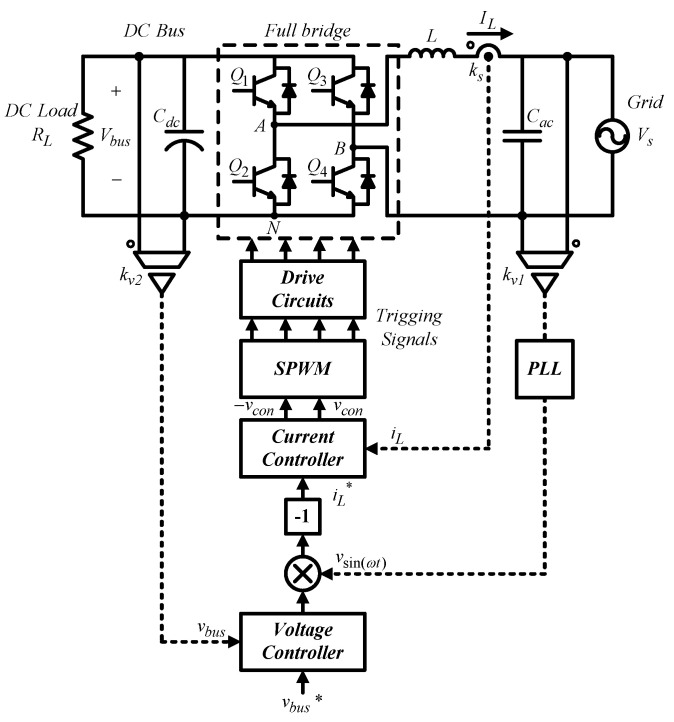
Grid-connected single-phase inverter control structure [29].

The grid-connected single-phase inverter adopts a dual-loop control scheme [29]. The outer loop adjusts DC bus voltage, and the inner loop adjusts inductor current. Sinusoidal PWM (SPWM) is used to generate trigger signals for the switches. Figure 6 shows the control structure, where A, B, and N are nodes; *V_bus_* represents DC bus voltage; *I_L_* represents inductor current; *V_s_* represents grid voltage; *k_v1_*, *k_v2_*, and *k_vs_* represent AC voltage, DC voltage, and current sensing scales, respectively; *v_bus_* and v_bus_* represent DC bus voltage feedback signal and its control command, respectively; *i_L_* and i_L_* represent inductor current feedback signal and its control command, respectively; *v_sin(__ωt)_* represent synchronization signal obtained by grid voltage through the phase-locked loop (PLL); and *v_con_* and *−v_con_* represent SPWM and its control voltage, respectively.

#### 3.1.1. Inner Loop Inductor Current Control

Using the switching functions of leg A and leg B, the voltage across the inductor can be derived from Figure 6:

(1) $$L\frac{dI_{L}}{dt}=k_{pwm}v_{con}-V_{s},\;k_{pwm}=\frac{V_{bus}}{v_{t}}.$$

**Figure 7:**
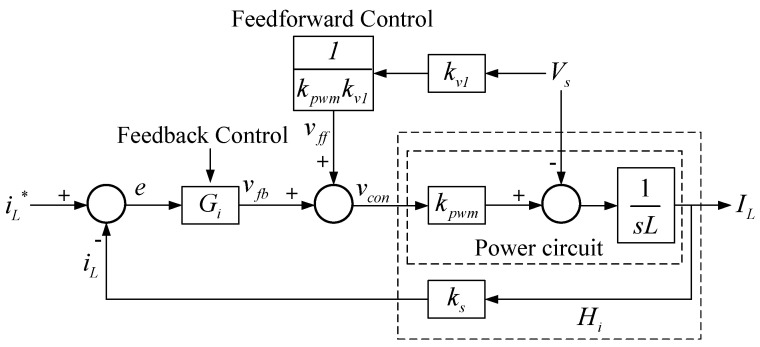
Inductor current control loop of the grid-connected single-phase inverter [29].

The control loop is shown in Figure 7, where the power circuit block is built according to (1), and *G_i_* adopts proportional (P) controller and feed-forward control in order to eliminate the disturbance in the loop caused by input voltage.

By letting *V_s_* = 0, we get:

(2) $$\frac{i_{L}}{i_{L}^{*}}=\frac{\frac{k_{1}k_{s}k_{pwm}}{sL}}{1+\frac{k_{1}k_{s}k_{pwm}}{sL}}=\frac{\frac{k_{1}k_{s}k_{pwm}}{L}}{s+\frac{k_{1}k_{s}k_{pwm}}{L}}=\frac{u_{i}}{s+u_{i}},$$

and the bandwidth of the current loop is:

(3) $$u_{i}=\frac{k_{1}k_{s}k_{pwm}}{L}.$$

As a result, after determining the bandwidth of the current loop, the gain of the P controller *k*_1_ can be obtained by (3). The bandwidth of a controller is generally designed to be between 1/4 and 1/10 times the switching frequency. In this case, the bandwidth is set to be 1/5 times the switching frequency, that is, 125,663.7 rad/s, and the gain of the P controller is 6.28.

#### 3.1.2. Outer Loop—DC Bus Voltage Controller

First, the transfer function of the control plant is derived. Considering unit input power factor (PF), the AC power on AC terminal is:

(4) $$\begin{array}{c} P_{ac}(t)=\hat{V_{s}}\sin(\omega t)\cdot \hat{I_{s}}\sin(\omega t)=\frac{\hat{V_{s}}\hat{I_{s}}}{2}-\frac{\hat{V_{s}}\hat{I_{s}}}{2}\cos\left(2\omega t\right)={\bar{P}}_{ac}+{\tilde{P}}_{ac2}, \end{array}$$

where ${\tilde{P}}_{ac2}$ causes second order ripple in DC voltage. Further derivation yields:

(5) $$\frac{V_{bus}}{\hat{I_{s}}}=\frac{\frac{k_{dc}}{C_{dc}}}{s+a},\;a=\frac{1}{R_{L}C_{dc}}.$$

**Figure 8:**
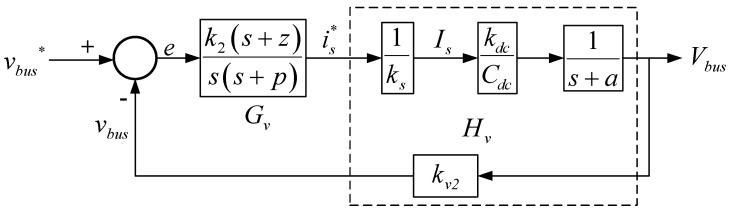
DC bus voltage control loop of the grid-connected single-phase inverter [29].

Figure 8 shows the control loop according to (5) based on the assumption that the current loop response bandwidth *u_i_* is much larger than the voltage loop bandwidth *u_v_*; as a result, the response of the current loop can be considered as 1 when analyzing voltage loop response.

According to Figure 8, the plant transfer function is:

(6) $$H_{v}\left(s\right)=\frac{k_{v2}k_{dc}}{k_{s}C_{dc}\left(s+a\right)}.$$

Since ${\tilde{P}}_{ac2}$ causes the second-order ripple in DC voltage, a type II controller is adopted, including a proportional–integral (PI) controller and a low pass filer (LPF), which attenuates the ripples, and thus reduces the distortion of the current command. The transfer function of a type II controller is:

(7) $$G_{v}\left(s\right)=PI*LPF=\frac{k_{2}\left(s+z\right)}{s\left(s+p\right)}.$$

The circuit specification of the single-phase inverter is shown as follows:

- Rated power *P*: 1 kVA;
- DC bus voltage *V_bus_*: 200 V;
- Grid rated voltage *V_s_*: 110 V_rms_;
- Grid voltage frequency *f_ac_*: 60 Hz;
- Power switching device: TPH3207WS
- Switching frequency *f_s_*: 100 kHz;
- Carrier amplitude *v_t_*: 5 V;
- AC voltage sensing scale *k_v1_*: 0.0062 V/V;
- DC voltage sensing scale *k_v2_*: 0.012 V/V;
- AC current sensing scale *k_s_*: 0.05 V/A;
- DC bus voltage variation percentage limit: 5%.

Considering the influence of the second order ripple of DC bus voltage on the stability of the controller, the gain crossover frequency of the voltage control loop is set at 16 Hz, and the pole and zero of the controller are selected: *p* = 180 rad/s, and *z* = 30 rad/s. These yield a plant crossover frequency gain of 1.2821 and controller crossover frequency gain of 0.0051. As a result, the gain of the controller required for compensation at the crossover frequency *k*_2_ is 154.1. The controller transfer function can be obtained as follows.

(8) $$G_{v}(s)=\frac{k_{2}(s+z)}{s(s+p)}=\frac{154.1(s+30)}{s(s+180)}.$$

**Figure 9:**
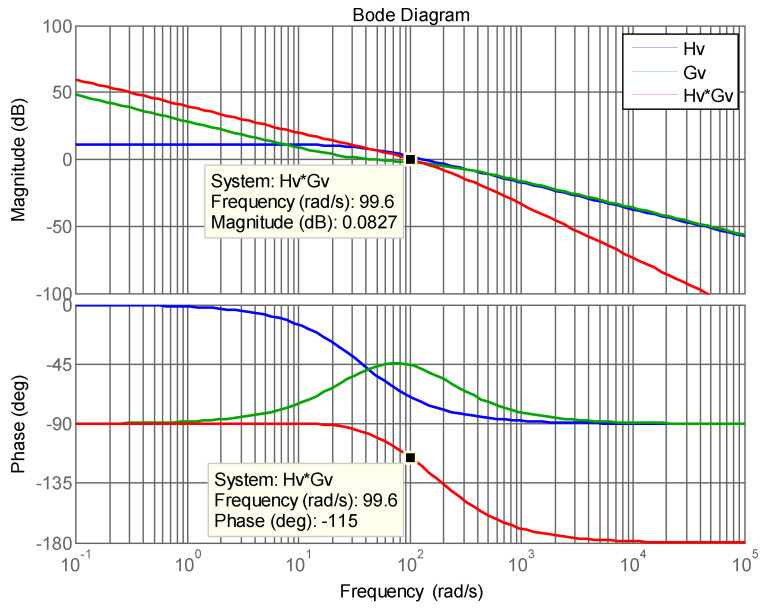
Open loop Bode plot of DC bus voltage control loop [29].

Figure 9 shows open-loop Bode plots of the plant and the controller. The phase margin is 65 degrees.

### 3.2. Quantitative Design of Controllers for Buck–Boost Converter

**Figure 10:**
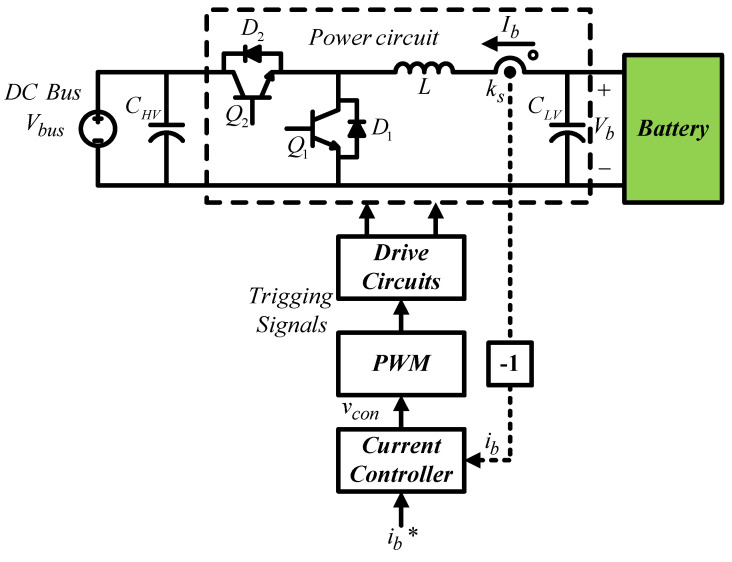
Buck–boost converter control structure [29].

The buck–boost converter adopts a single loop to control the inductor current. The control structure is shown in Figure 10, where *V_bus_* represents DC bus voltage; *I_b_* represents inductor current; *k_s_* represents current sensing scale; *i_b_* and i_b_^*^ represent inductor current feedback signal and its control command, respectively; and *v_con_* represents PWM control voltage.

The controller design is based on the following design specification:

- Rated power *P*: 1 kW;
- Battery voltage *V_b_*: 96 V;
- DC bus voltage *V_bus_*: 200 V;
- Power switching device: TPH3207WS
- Switching frequency *f_s_*: 100 kHz;
- Carrier amplitude *v_t_*: 5 V;
- Current sensing scale *k_s_*: 0.05 V/A;
- Inductor current ripple percentage limit: 20% (output current).

#### Single Loop Inductor Current Controller

Using the mathematical derivation of inductor voltage, we get:

(9) $$L\frac{d\hat{i_{b}}(t)}{dt}=D\hat{v_{bus}}(t)+V_{bus}\hat{d}(t)-\hat{v_{b}}(t).$$

**Figure 11:**
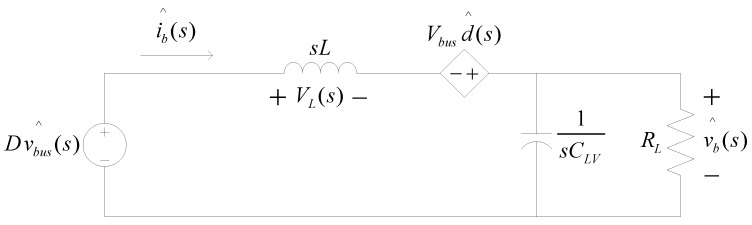
Buck–boost converter small signal model [29].

After Laplace transform, the small signal model can be presented as in Figure 11.

Letting $\hat{v_{bus}}(s)=0$, we get

(10) $$\frac{\hat{i_{b}}(s)}{\hat{d}(s)}=\frac{V_{bus}}{\left[sL+\frac{R_{L}}{1+sC_{LV}R_{L}}\right]}=\frac{V_{bus}(1+sC_{LV}R_{L})}{sL(1+sC_{LV}R_{L})+R_{L}}=\frac{V_{bus}}{L}\cdot \frac{s+\frac{1}{R_{L}C_{LV}}}{s^{2}+\frac{1}{R_{L}C_{LV}}s+\frac{1}{LC_{LV}}}.$$

**Figure 12:**
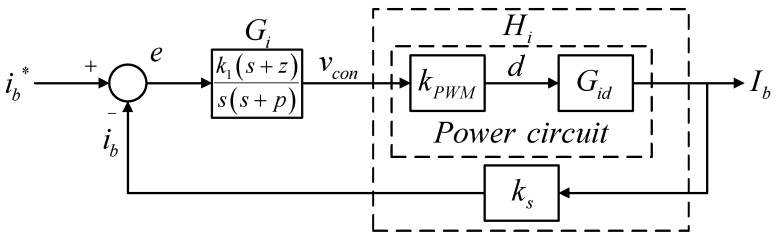
Buck–boost converter inductor current control loop [29].

Figure 12 shows the inductor current control loop according to (10), in which a Type II controller is adopted. In Figure 12, the transfer function of control plant can be expressed in (11).

(11) $$H_{i}(s)=k_{PWM}\cdot G_{id}\cdot k_{s}=\frac{1}{v_{t}}\times \frac{\hat{i_{b}}(s)}{\hat{d}(s)}\times k_{s}=\frac{k_{s}\cdot V_{bus}}{v_{t}\cdot L}\cdot \frac{s+\frac{1}{R_{L}C_{LV}}}{s^{2}+\frac{1}{R_{L}C_{LV}}s+\frac{1}{LC_{LV}}},$$

where *R_L_* represents load resistance. Substituting related design specification and parameters into (11) yields:

(12) $$H_{i}(s)=\frac{10000s+1.302\times 10^{8}}{s^{2}+1.302\times 10^{4}s+5\times 10^{8}}.$$

Next, the crossover frequency needs to be selected. In this case, the crossover frequency is set at 1/10 times the switching frequency, which is 62,832 rad/s, and calculation yields K factor value: 3.5291, and thus the zero and pole of the controller are 17,804 rad/s and 221,740 rad/s, respectively. Therefore, the controller transfer function is obtained:

(13) $$G_{i}\left(s\right)=\frac{k_{1}\left(s+z\right)}{s\left(s+p\right)}=\frac{1.2244\times 10^{6}\left(s+17804\right)}{s\left(s+221740\right)}.$$

### 3.3. Quantitative Design of Controllers for PV-Boost Converter

**Figure 13:**
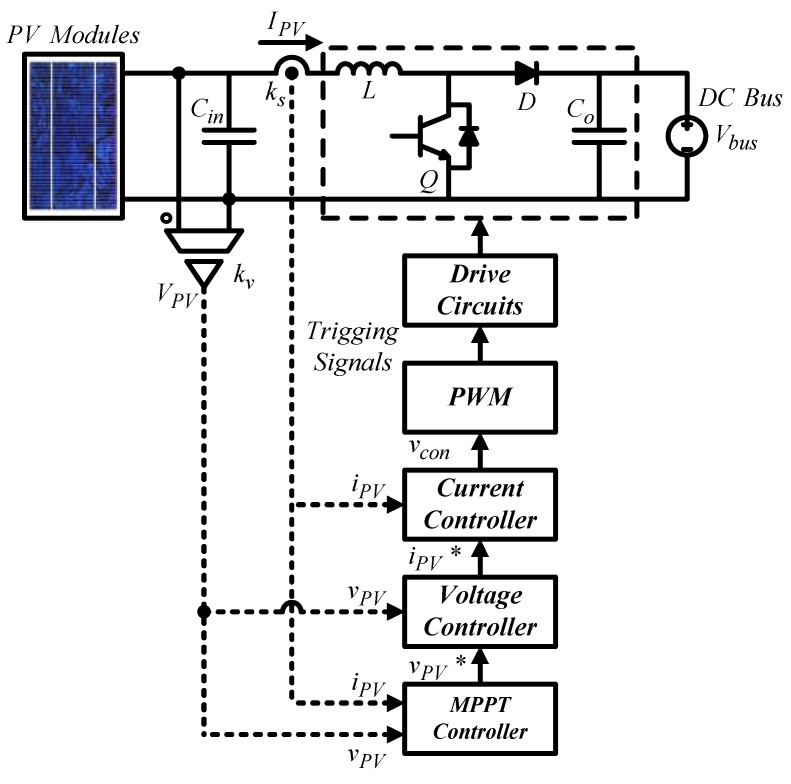
PV-boost converter control structure.

The PV-boost converter adopts dual-loop control scheme, where the outer loop adjusts the output voltage of the PV module, and the inner loop adjusts inductor current. Figure 13 shows the control structure, where *V_PV_* represents PV module output voltage; *I_PV_* represents PV module output current (inductor current); *V_bus_* represents DC bus voltage; *k_s_* and *k_v_* represent current and voltage sensing scales, respectively, *v_PV_* and v_PV_^*^ represent output voltage feedback signal and its control command of the PV module, respectively; *i_PV_* and i_PV_^*^ represent output current feedback signal and its control command of the PV module, respectively; and *v_con_* represents PWM control voltage.

The design specification of the PV-boost converter is as follows:

- Rated power *P*: 600 W;
- Input voltage *V_PV_*: 60~100 V;
- DC bus voltage *V_bus_*: 200 V;
- Power switching device: TPH3207WS
- Switching frequency *f_s_*: 100 kHz;
- Carrier amplitude *v_t_*: 5 V;
- Voltage sensing scale *k_v_*: 0.024 V/V;
- Current sensing scale *k_s_*: 0.24 V/A;
- Inductor current ripple percentage limit: 20% (output current).

#### Inner Loop-Inductor Current Control

For a boost converter, the operation can be divided into two circuit states according to whether the switch Q is on or off in a single switching cycle: state 1 is when Q is on, and D is off; and state 2 is when Q is off, and D is on. Current transfer function can be derived:

(14) $$\frac{\hat{i_{PV}}(s)}{\hat{d}(s)}=\frac{V_{bus}}{sL}.$$

**Figure 14:**
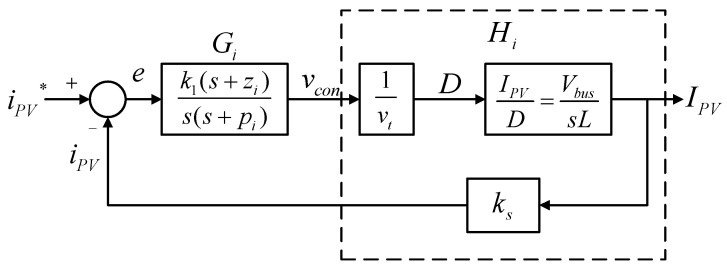
PV-boost converter inductor current control loop.

Figure 14 shows inductor current control loop according to (14).

The transfer function of the control Plant *H_i_* can be derived according to Figure 14:

(15) $$H_{i}(s)=\frac{1}{v_{t}}\times \frac{V_{bus}}{sL}\times k_{s}=\frac{V_{bus}\cdot k_{s}}{v_{t}\cdot sL}.$$

Next, the crossover frequency needs to be selected. Here, it is set at 1/10 times the switching frequency to obtain the controller expressed in (16):

(16) $$G_{i}\left(s\right)=\frac{k_{1}\left(s+z_{i}\right)}{s\left(s+p_{i}\right)}=\frac{7.213\times 10^{5}\left(s+16836\right)}{s\left(s+234495\right)}.$$

**Figure 15:**
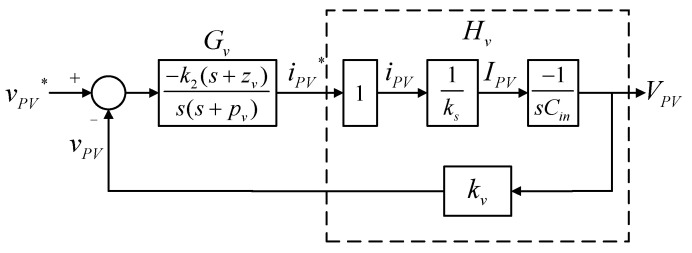
PV module output voltage control loop.

Based on Figure 13, the output voltage control loop can be constructed as shown in Figure 15. The voltage control loop crossover frequency is set at 1/5 times the crossover frequency of the current control loop, that is, 12,566 rad/s.

The transfer function of *H_v_* can be derived from Figure 15, where the negative sign is offset by the controller *Gv*:

(17) $$H_{v}(s)=1\times \frac{1}{k_{s}}\times \frac{1}{sC_{in}}\times k_{v}=\frac{k_{v}}{k_{s}\cdot sC_{in}}.$$

After derivation, the gain of the plant at the crossover frequency *Gain_H_* and its phase *Angle_H_* can be found as 0.7958 and −90°, and thus the phase boost required by the controller at the crossover frequency is 60°. Then, the K factor is calculated to be 3.7321, and thus the zero and pole are calculated to be 3367 rad/s and 46,898 rad/s, respectively. Consequently, the gain that the controller needs for compensation at the crossover frequency is 58,932. As a result, we get the controller transfer function as follows.

(18) $$G_{v}\left(s\right)=\frac{k_{2}\left(s+z_{v}\right)}{s\left(s+p_{v}\right)}=\frac{58932\left(s+3367\right)}{s\left(s+46898\right)}.$$

## 4. Operating Mode Simulation and Analysis

**Figure 16:**
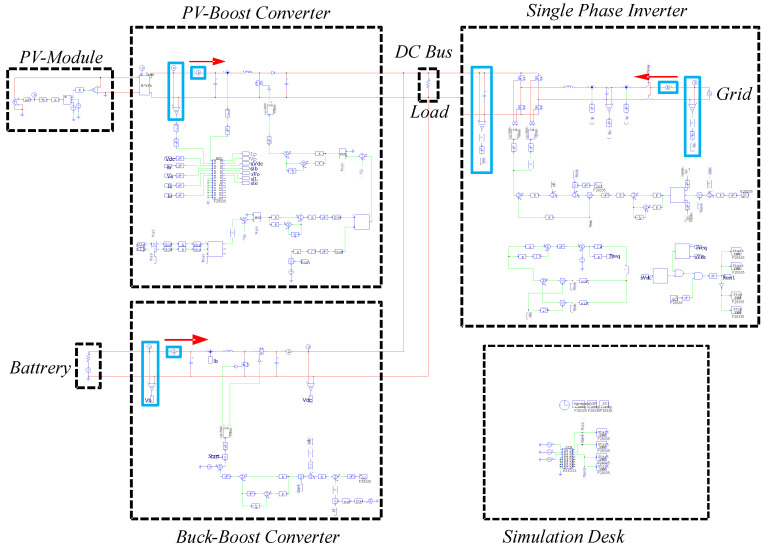
The PSIM model of the proposed grid-connected RE-based power generation system.

In this paper, the software, PowerSIM (PSIM), is used for obtaining results of various scenario simulations, which can be used to compare the results from the hardware implementation and to verify the correctness of the designed controllers and the feasibility of the operating modes. The complete simulation configuration is shown in Figure 16.

### 4.1. Operating Mode 1: PV Power Smoothing Mode

**Figure 17:**
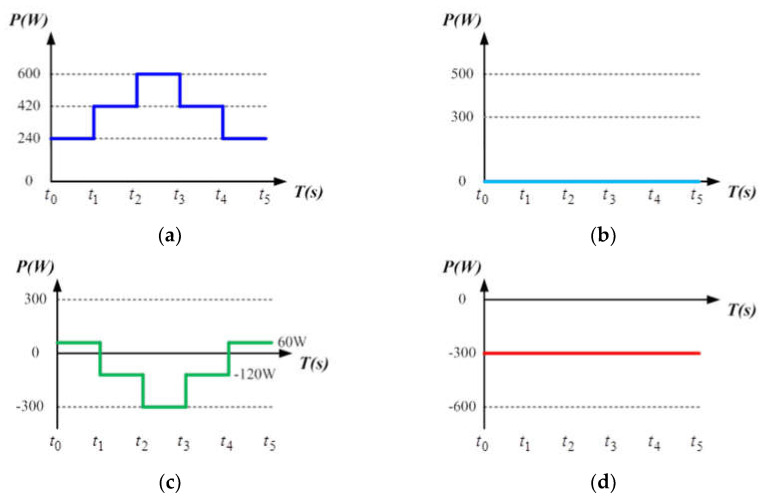
Power variations of: (**a**) PV generation; (**b**) load; (**c**) battery; and (**d**) grid in mode 1.

In this mode, the battery’s SOC is assumed between 25 ± 1% and 90 ± 1%, which is a safe operating range for most battery banks to allow that the charge/discharge can be arbitrarily carried out in an appropriate range. To construct a severe power fluctuation scenario, zero load power demand is assumed and full PV generation with MPPT control is activated. Considering the impact of RE output power fluctuations on the grid, the system adopts an instant charge/discharge function for power smoothing based on the concept of power dispatch. In this mode, PV generation is divided into three intervals within a day: early morning or evening (low irradiance), noon (high irradiance), and other periods (medium irradiance), without considering other factors that affect sunlight, such as cloud shading, and the temperature is fixed at 25 °C. Irradiance values of 400 W/m^2^, 700 W/m^2^, and 1000 W/m^2^ correspond to PV module maximum power points of 240 W, 420 W, and 600 W, respectively. Assuming that the power injected to the grid is fixed at 300 W, if PV power is greater than 300 W, the excess power will be stored in the battery; if PV power is less than 300 W, the battery is discharged to make up the required power. The variations in PV power generation, load, battery, and grid are shown in Figure 17.

**Figure 18:**
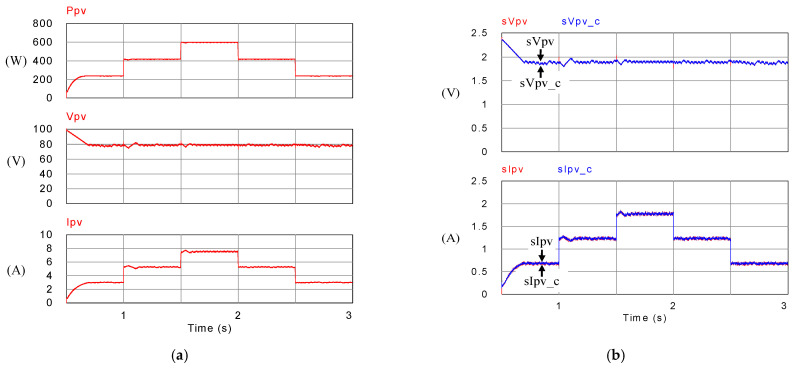
Mode 1 PV module (**a**) output power, voltage, and current; and (**b**) feedback and control commands of output voltage and current.

**Figure 19:**
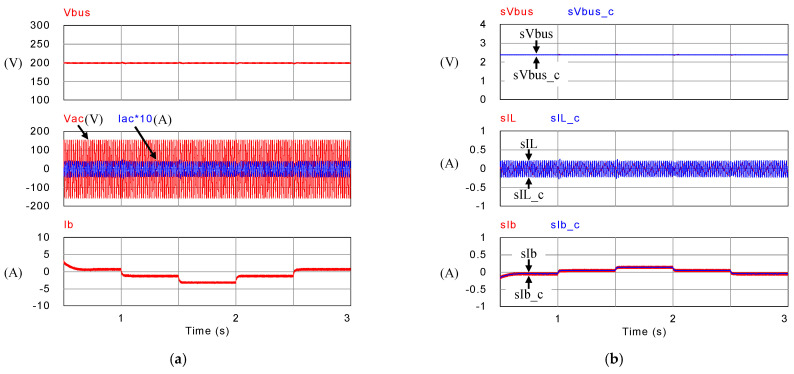
Mode 1 (**a**) DC bus voltage, grid voltage and current, and battery current; and (**b**) feedback and control commands of DC bus voltage, inductor current, and battery current.

**Figure 20:**
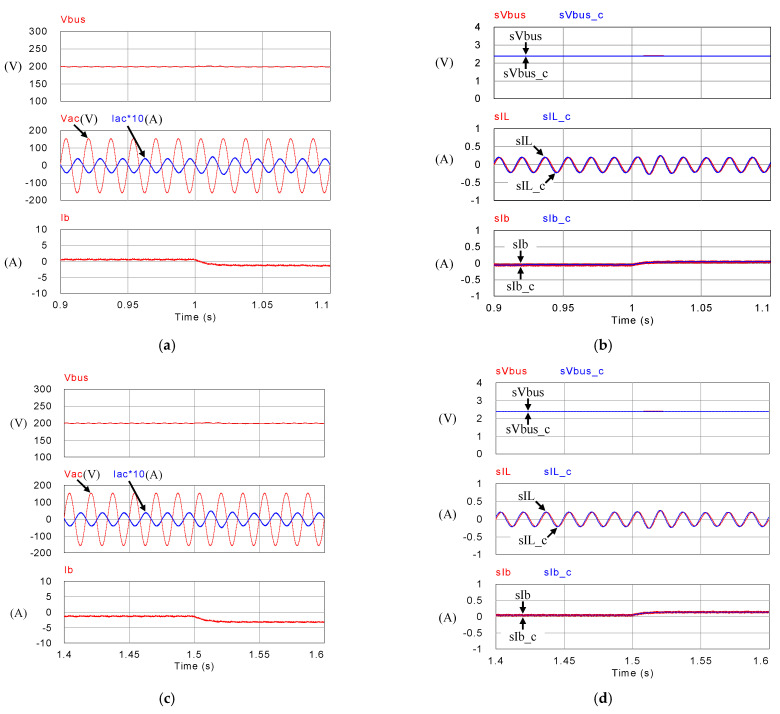
Detailed view of (**a**) Figure 19a at t_1_, (**b**) Figure 19b at t_1_, (**c**) Figure 19a at t_2_, and (**d**) Figure 19b at t_2_.

**Figure 21:**
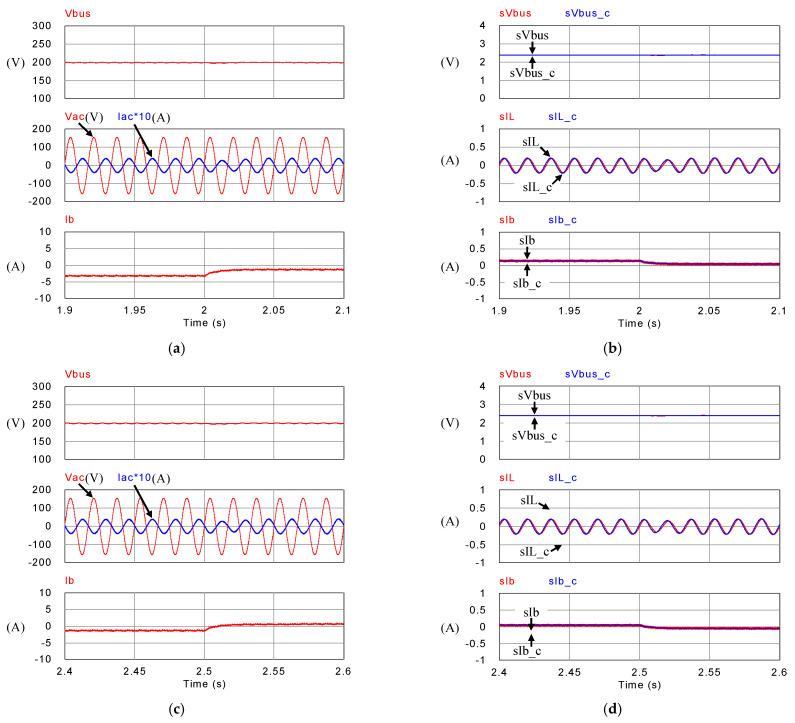
Detailed view of (**a**) Figure 19a at t_3_, (**b**) Figure 19b at t_3_, (**c**) Figure 19a at t_4_, and (**d**) Figure 19b at t_4_.

Figure 18, Figure 19, Figure 20 and Figure 21 show simulation waveforms of operating mode 1. For easy observation of DC bus voltage at the instant of irradiance change and grid power smoothing, Figure 20 and Figure 21 are the detailed view at switching points t_1_~t_4_ in Figure 19. It can be observed that the overshoots of DC bus voltage at t_1_ and t_2_ are both 1.5 V (0.75%), and the undershoots at t_3_ and t_4_ are both 1.2 V (0.6%), which all meet the specified 5% limitation. According to Figure 18a, as generation condition changes, the PV module continues to output at maximum power. Additionally, in the overall simulation process, regardless of the variation in PV power, the system feeds stable power to the grid. This also shows that the system stably maintains DC bus voltage. Additionally, the battery charge/discharge command is adjusted according to the variation in PV generation to smooth out the power fluctuation so that the back feed power can be kept stable, and thus the goal of power smoothing is achieved.

### 4.2. Operating Mode 2: Peak Power Regularion Mode

**Figure 22:**
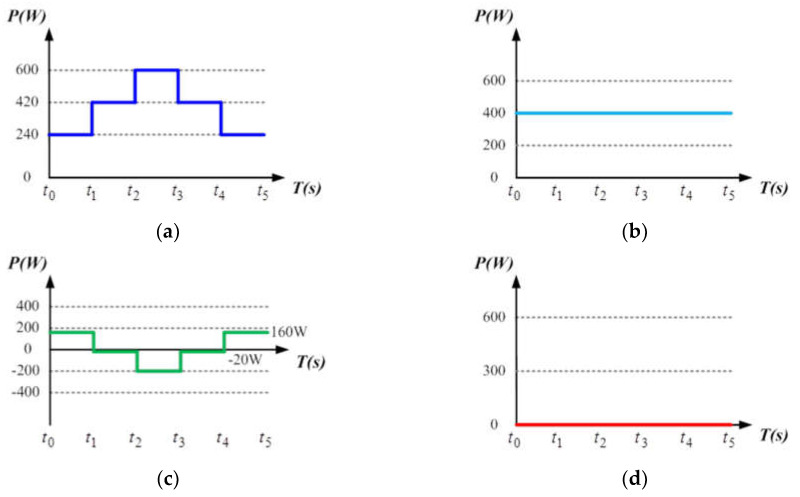
Power variation of: (**a**) PV generation; (**b**) load; (**c**) battery; and (**d**) grid in mode 2.

In this mode, the PV conditions are similar to mode 1, except there is a constant load power demand of 400 W. The power variation of PV, load, battery, and grid are graphically shown in Figure 22.

**Figure 23:**
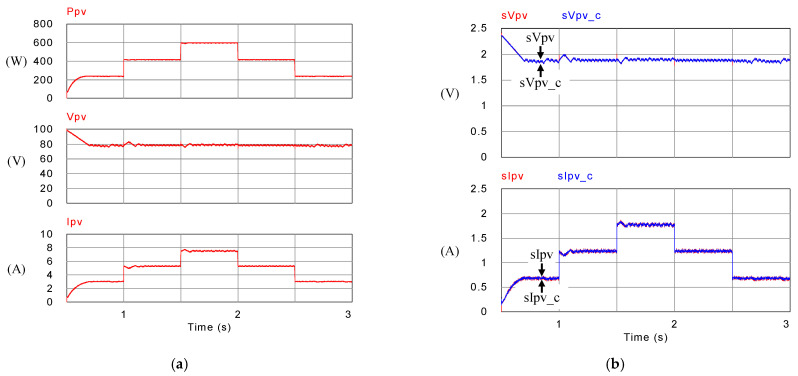
Mode 2 PV module (**a**) output power, voltage, and current; and (**b**) feedback and control commands of output voltage and current.

**Figure 24:**
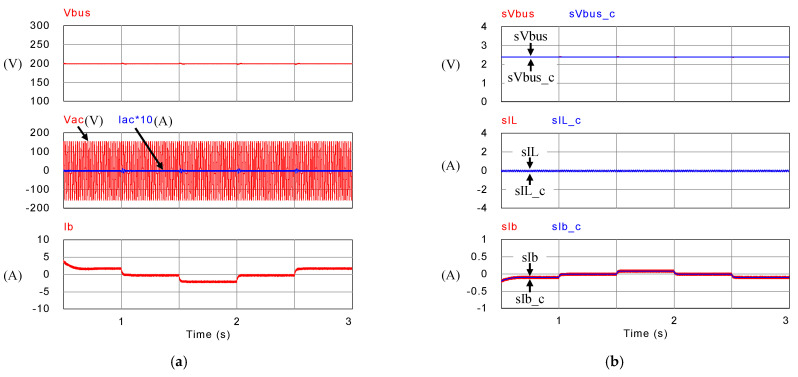
Mode 2 (**a**) DC bus voltage, grid voltage and current, and battery current; and (**b**) feedback and control commands of DC bus voltage, inductor current, and battery current.

**Figure 25:**
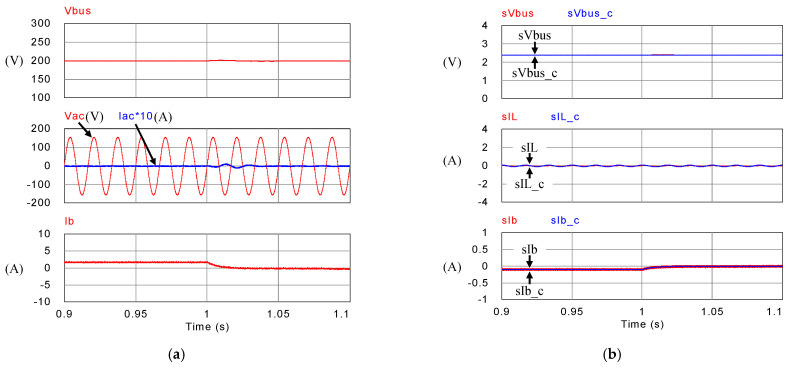

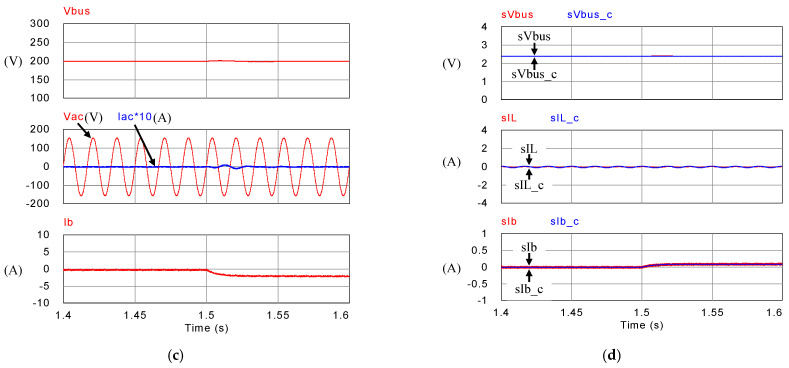
Detailed view of (**a**) Figure 24a at t_1_, (**b**) Figure 24b at t_1_, (**c**) Figure 24a at t_2_, and (**d**) Figure 24b at t_2_.

**Figure 26:**
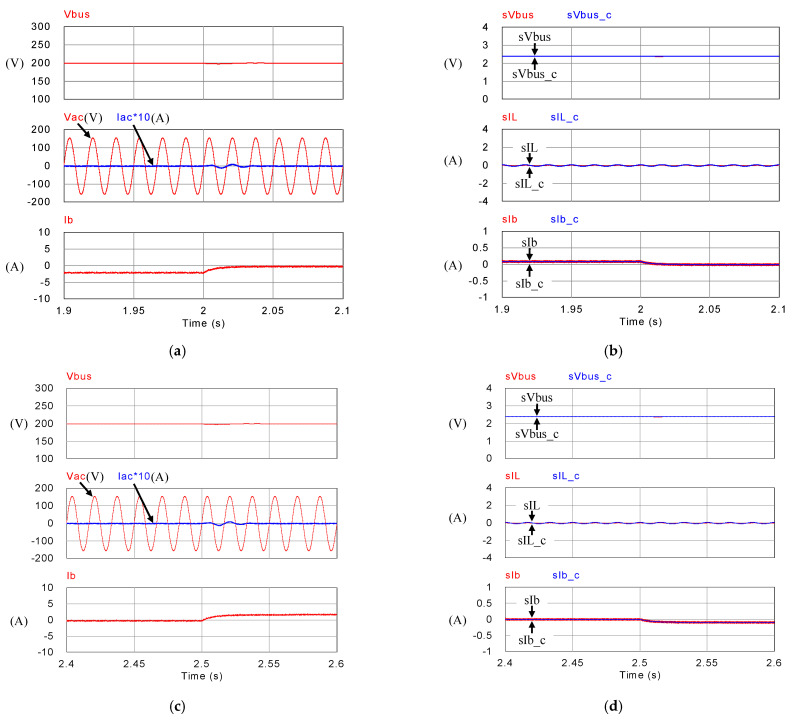
Detailed view of (**a**) Figure 24a at t_3_, (**b**) Figure 24b at t_3_, (**c**) Figure 24a at t_4_, and (**d**) Figure 24b at t_4_.

Figure 23, Figure 24, Figure 25 and Figure 26 show simulation waveforms of peak power regulation at 400 W load demand. It can be observed that the battery compensates for insufficient PV generation and absorbs excessive power generation. Figure 25 and Figure 26 are the detailed view at switching points t_1_~t_4_ in Figure 24, respectively. The overshoot and undershoot of DC bus voltage at all switching points are 1.7 V (0.85%), which meet the specified 5% limit on DC bus voltage variation. During peak periods, the system can immediately respond with proper charge/discharge commands. PV generation and battery are used to meet the load power demand to avoid the use of expensive power from the grid. As a result, the objective of peak power regulation is achieved.

With the above simulation results, it can be verified that the grid-connected RE-based power generation system developed in this paper can be operated in the two operation modes based on various application scenarios.

## 5. Hardware Implementation and Test Results

**Figure 27:**
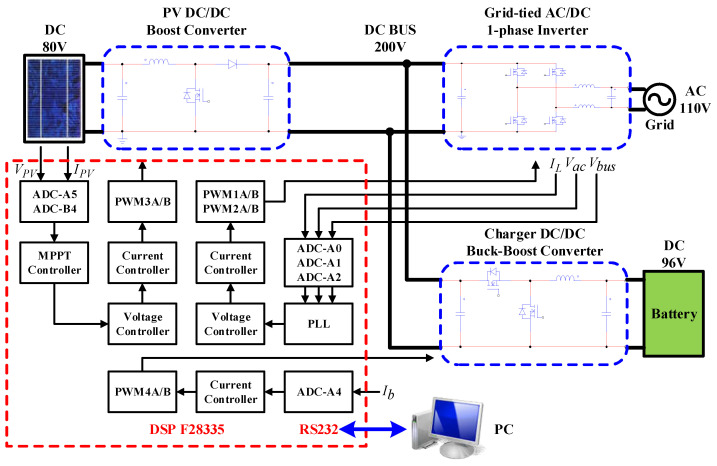
The full digital controlled small-capacity hardware experimental system.

To further verify the performance of proposed control schemes, a 1 kVA small-capacity experimental system integrating three GaN-based power converters with full digital control schemes is constructed in this paper. The control core of the system adopts TI’s DSP TMS320F28335 to simplify hardware circuits. The PWM control signals and A/D converters required for the converters adopt six sets of A/D converters and four sets of PWM signals. The full digital controlled hardware experimental system proposed in this paper is shown in Figure 27.

**Figure 28:**
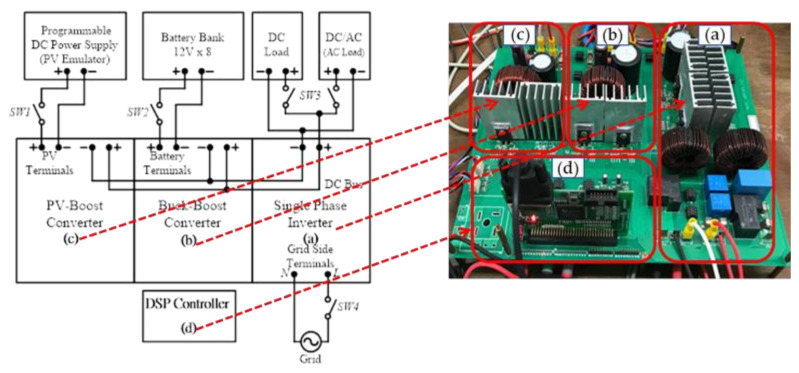
Block diagram of the proposed hardware configuration and the photo of the constructed circuit for experimental tests.

The block diagram of the complete hardware configuration of the proposed grid-connected RE-based power generation system and the photo of the constructed GaN based power converters for carrying out experimental tests are shown in Figure 28.

### 5.1. Operating Mode 1: PV Power Smoothing Mode

**Figure 29:**
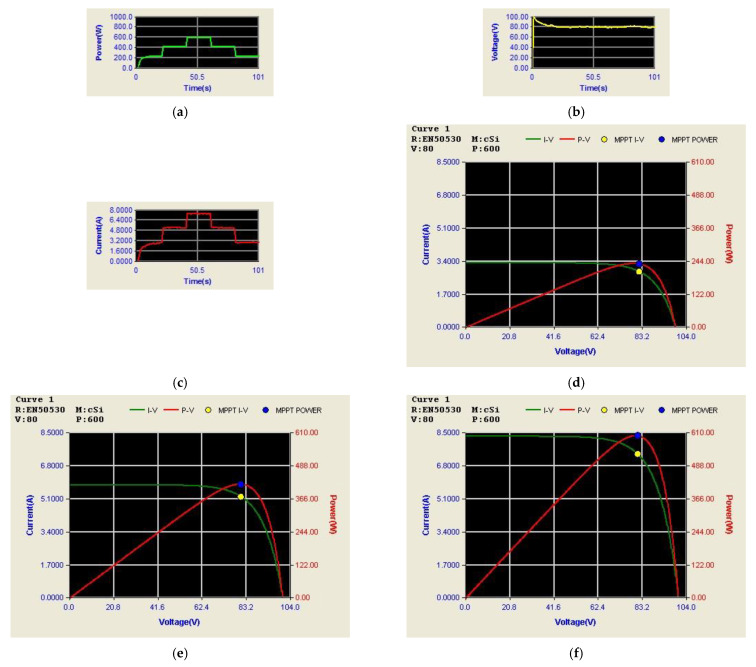
Results in Mode 1: PV output (**a**); power; (**b**) voltage; (**c**) current; P-V/V-I characteristic curves and MPPT at the irradiance of (**d**) 400 W/m^2^; (**e**) 700 W/m^2^; and (**f**) 1000 W/m^2^.

Figure 29 shows the arrangement of PV module output power, voltage, and current, as well as P-V/V-I characteristic curves and MPPT under different irradiances. It can be observed that under the conditions of irradiance of 400 W/m^2^, 700 W/m^2^, and 1000 W/m^2^ and a constant temperature of 25 °C, the system can correctly reach to the maximum power point of 240 W, 420 W, and 600 W, respectively.

**Figure 30:**
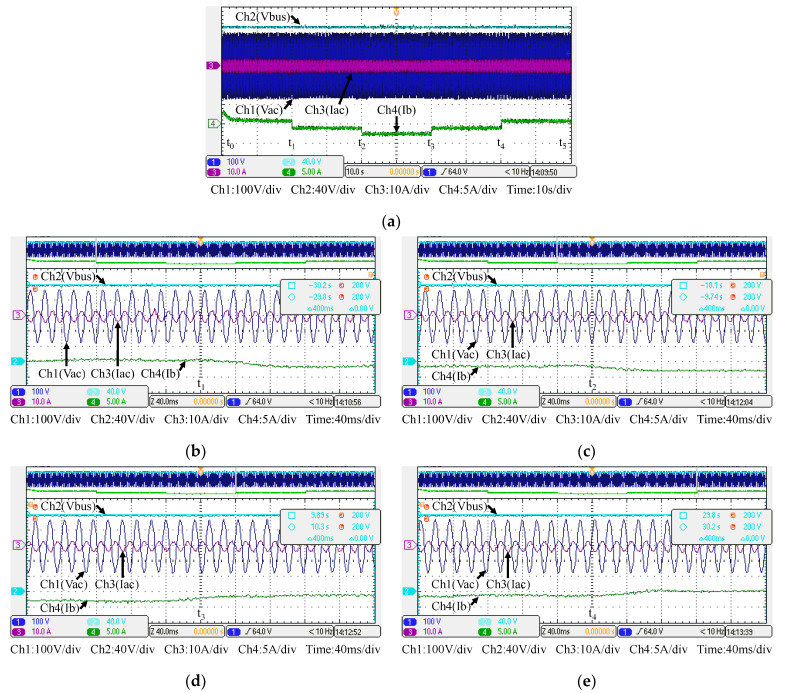
Results in Mode 1: (**a**) DC bus voltage, grid voltage and current, and battery current, and its detailed view at: (**b**) t_1_; (**c**) t_2_; (**d**) t_3_; and (**e**) t_4_.

Figure 30 shows the measured voltage/current waveforms of the system. Figure 30a shows the result of power smoothing control: DC bus voltage is stably controlled to the target value of 200 V with less than 1% deviation at all maximum power points. Figure 30b–e show the detailed view at switching points t_1_~t_4_ in Figure 30a, respectively. The overall experimental test results are quite similar to the results obtained in simulation. Based on the quantitative measured results, the error in the real power regulation is less than 2% at all maximum power points, and thus the function of PV power smoothing is verified.

### 5.2. Operating Mode 2: Peak Power Regulation Mode

**Figure 31:**
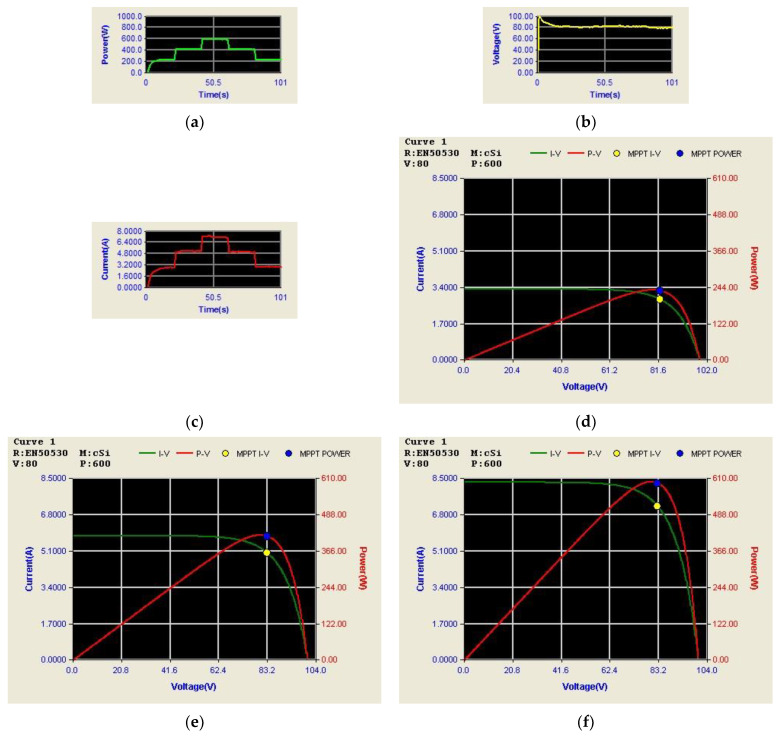
Results in Mode2: PV output; (**a**) power; (**b**) voltage; and (**c**) current; and P-V/V-I characteristic curves and MPPT at the irradiance of (**d**) 400 W/m^2^; (**e**) 700 W/m^2^; and (**f**) 1000 W/m^2^.

**Figure 32:**
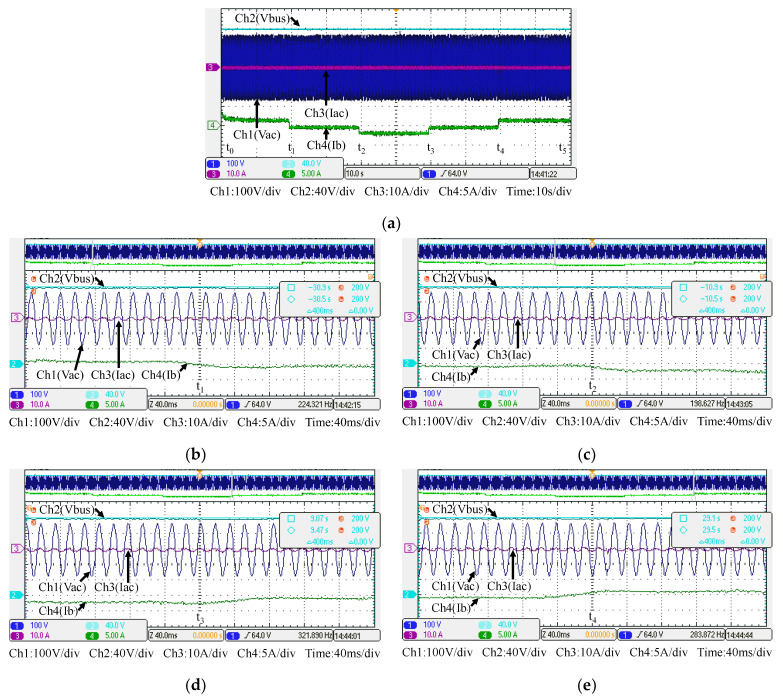
Results in Mode 2: (**a**) DC bus voltage, grid voltage and current, and battery current, and its detailed view at: (**b**) t_1_; (**c**) t_2_; (**d**) t_3_; and (**e**) t_4_.

Figure 31 shows the arrangement of system conditions, i.e., PV module output power, voltage, and current, as well as P-V/V-I characteristic curves and MPPT under different irradiance. Comparing these figures with simulation results, it can be confirmed that the performance of the developed PV-boost converter is well in line with expectations. Figure 32a shows voltage and current waveforms of the grid, the battery, and DC bus. Figure 32b–e show a detailed view of Figure 32a at switching points t_1_~t_4_, respectively. Based on the results presented in this subsection, the performance is inconsistent with the simulation results.

Figure 32 shows the measured voltage/current waveforms of the system. Figure 32a shows the result of peak power regulation, in which the DC bus voltage is stably controlled to the target value of 200 V with less than 1% deviation. Figure 32b–e shows the detailed view at switching points t_1_~t_4_ in Figure 32a, respectively. The overall implementation test results are almost identical to that obtained in the simulation studies. As can be seen in Figure 32a, the grid current is well regulated at zero, thus the peak power regulation function is fully achieved.

## 6. Discussion

In response to the global net-zero trend and the rapid changes in the international energy environment, the world is at a critical stage of energy transformation. Renewable energy-based power generation, smart grid, energy storage, advanced power converter systems, and the state-of-art system integration technologies became the key driving forces for creating a robust electric power grid and new smart energy industries. The development and verification of advanced power converter technologies and control schemes are urgently required. It is well accepted that power converter systems can benefit from integration technologies in many ways, such as reducing size, improving reliability, and reducing hardware costs. In recent years, with the continuous development of power semiconductor devices, especially the emergence of WBG switching devices, such as GaN high-electron mobility transistors (HEMTs) and silicon carbide (SiC) metal oxide semiconductor field-effect transistors (MOSFETs) offer huge potential for outperforming conventional silicon (Si) devices in terms of higher breakdown voltage, temperature capability, switching speed, and lower conduction losses. If these superior characteristics are smartly utilized, it is possible to upgrade the features of power electronic devices and the performance of existing power conversion systems. In this aspect, advanced integration technologies and system verification in practical engineering applications are needed. In the aspect of developing advanced power converter technology on WBG switching devices and system integration technologies for renewable power generation systems, this paper makes the following contributions to the field:

1. This is the first time that a complete design case demonstrating three GaN-based power converters with different control functions integrated with a single DSP chip to achieve a reliable, flexible, cost-effective, and multifunctional power interface for renewable power generation systems;
2. In this paper, three GaN-based power converters with distinct control functions are smartly integrated with an advanced digital control scheme to achieve a flexible power interface for the grid-tied renewable power generation system embedded with a battery energy storage unit. Advantages of the proposed control scheme, including high control flexibility and high system reliability, are demonstrated via case simulations and hardware tests in two typical operating modes, i.e., PV power smoothing control and peak power regulation modes;
3. This paper proposes an advanced power control scheme that efficiently integrates renewable energy-based power generation, an energy storage system, and various loads on a common DC bus. Based on the results obtained from the simulation and hardware tests, the real-time power balance and management functions of the renewable power generation system are automatically achieved with the proposed fast-response, dual-loop DC bus voltage controller;
4. In this paper, a systematic design process with detailed design steps of various controllers is presented. This is a great merit for researchers and engineers in the power engineering field.

## 7. Conclusions

Due to the intermittent and unpredictable nature of RE, hybrid power generation systems that integrate multiple RE sources and energy storage devices became the focus of research in recent years. Since REs have the problems of low energy conversion efficiency and multiple operation modes, it is significant to improve the efficiency of the power converters with high-performance WBG semiconductor-based power converters and optimize the system performance using advanced control schemes to integrate various power converters in the system. It is well accepted that power converter systems can benefit from integration technologies in many ways, such as reducing size, improving reliability, and reducing hardware costs. This paper demonstrated a successfully designed grid-connected hybrid RE-based power generation system embedded with an energy storage unit and digitally integrated with multiple high-performance WBG-based power interfaces. This paper firstly reviewed related papers on hybrid RE-based power generation systems and power smoothing technologies and then proposed the system configuration and operating modes of a grid-connected RE-based power generation system considering practical application scenarios. The circuit configuration and mathematical model derivation of the three functional GaN-based power converters used in the proposed system and the quantitative design of various controllers were explained in detail and verified with computer simulations. In this paper, two typical operating scenario cases, i.e., PV power smoothing mode and peak power regulating mode were verified with a 1-kVA small-capacity hardware experimental system. From both simulation and hardware test results of the two operating modes, it can be verified that the proposed hybrid RE-based power generation system integrated with WBG-based power interfaces and the designed power flow control schemes are correct and feasible.

## Data Availability

No new data were created or analyzed in this study. Data sharing is not applicable to this article.
